# Relationship between peer victimization and adolescent internet addiction: self-esteem as a mediator and teacher-student relationship as a moderator

**DOI:** 10.3389/fpsyg.2026.1762386

**Published:** 2026-05-14

**Authors:** Yujie Wang, Cheng Sun, Honglin Wu, Yuhe Shan, Yuxin Wang, Yajie Zhang, Siqi Wu, Yanfei Lu, Chenchen Xu

**Affiliations:** 1 Fourth Clinical Medical College, Nanjing Medical University, Nanjing, Jiangsu, China; 2 School of Public Health, Nanjing Medical University, Nanjing, Jiangsu, China; 3 Nantong Tianjiabing Middle School of Jiangsu Province, Nantong, Jiangsu, China

**Keywords:** internet addiction, moderating and mediating factors, peer victimization, self-esteem, teacher–student relationship

## Abstract

**Background:**

Adolescent Internet addiction (IA) has emerged as a significant mental health concern. Peer victimization (PV) is a prevalent risk factor in school settings, necessitating further examination of its impact mechanism on IA. There is a notable gap in systematic exploration of the interaction between individual traits, such as self-esteem (SE), and environmental resources in shaping IA.

**Purpose:**

This study explores the relationship between PV and adolescent IA, elucidates the mediating role of self-esteem (SE), and assesses the moderating influence of the teacher-student relationship (TSR). A moderated mediation model is established to uncover the mechanisms linked to the emergence of IA.

**Methods:**

An opportunity sampling method was employed to select 1,162 students from six high schools in Jiangsu Province, China. Data were gathered utilizing the Multi-Dimensional Peer Victimization Scale (MPVS), Rosenberg Self-Esteem Scale (RSES), Teacher-Student Relationship Scale (TSRS), and the Chinese Internet Addiction Scale-Revised (CIAS-R). Correlation analysis, Bootstrap mediation testing, and moderated mediation effect analysis were employed for statistical analysis.

**Results:**

PV significantly and positively predicted adolescent IA (*r* = 0.288, *p* < 0.001). SE partially mediated the relationship between PV and IA, accounting for 33.5% of the total effect (effect value = 0.336, 95% CI [0.251, 0.424]). TSRs significantly moderated the first half of the mediation path (PV × TSR: *β* = −0.018, *p* < 0.001) and the direct effect of PV on IA (*β* = 0.014, *p* < 0.05). A positive TSR significantly buffered the negative impact of PV on SE and the promoting effect of PV on IA compared to a poor TSR.

**Conclusion:**

PV indirectly positively associates with the risk of IA by negatively linking with adolescents’ SE, whereas positive TSR can effectively mitigate this detrimental pathway. This study’s findings establish a theoretical framework for creating targeted intervention strategies for adolescent IA, indicating that schools can alleviate the adverse effects of PV by improving SE levels and reinforcing the teacher-student support system. For example, schools could regularly conduct extracurricular activities that promote high SE, offer counseling services for students with low SE, and implement teacher training focused on improving TSR.

## 1 Introduction

### 1.1 Background

IA denotes a significant psychological and physiological reliance on the Internet (Vally and Allaghi, 2019), characterized by an inability to regulate Internet use, often manifesting as uncontrolled impulses, tolerance, and withdrawal symptoms (Zhao and Jin, 2023). *The 5th National Survey on Internet Use Among Minors* indicates that the number of minor Internet users in China has surpassed 193 million, with Internet access at 97.2%. Among them, the Internet usage rate for adolescents in middle and high school exceeds 99% (China Youth Net, 2023). A previous research indicates that the prevalence of IA among Chinese adolescents has reached 14.1%, exhibiting a continuous upward trend (Zhai et al., 2020). Upon diagnosis of IA, adolescents may experience significant adverse effects on their physical and mental health, along with disruptions to their typical social development. At the individual level, IA can hinder adolescents’ interpersonal skills, contributing to emotional disorders such as depression and anxiety, which may ultimately lead to adverse behaviors like school avoidance and self-harm (Yusuf et al., 2022). At the societal level, IA weakens adolescents’ moral understanding and legal awareness, resulting in personality distortion and confusion regarding social roles (Guo and You, 2024). Given the substantial population of adolescent Internet users and the detrimental impacts of IA, it is essential to investigate the underlying factors and mechanisms associated with adolescent IA. This exploration will offer theoretical support for the formulation of effective intervention strategies.

### 1.2 PV and IA

Peer relationships are interpersonal ties formed and cultivated through encounters among individuals of comparable age or psychological development levels (Berndt et al., 1999, p16). According to the notion of group socialization, peer groups constitute the principal social environment for individuals (Reis-Dennis, 2020). Given their distinctive status, adolescents engage more frequently with peers than with parents (Larson and Richards, 1991), rendering peer connections more crucial for their physical and mental development. Multiple studies have demonstrated that positive peer interactions are inversely associated with adolescent IA. A survey conducted online with 782 Chinese adolescents revealed that increased trust among peers correlated with a diminished likelihood of IA, while decreased peer trust constituted a significant risk factor for IA (Feng et al., 2025). A study conducted by Li et al. (2024) with 1,992 Chinese high school students identified a substantial positive link between PV and IA: increased severity of PV corresponded with a heightened probability of IA. Teng et al. (2020) employed a four-year longitudinal tracking strategy to survey 3,079 adolescents, including high school students, at four intervals. The findings indicated that swift enhancement in peer support significantly predicted the expedited decrease of IA symptoms, suggesting that positive peer relationships currently can consistently mitigate future IA tendencies.

The Compensatory Internet Use Theory asserts that persons who face adverse life events suffer an increase in negative emotions. These emotions compel individuals to utilize the Internet as an escape from current reality, aiming to satisfy unmet wants that remain unfulfilled in real life, thereby heightening the probability of developing IA (Yin and Shen, 2024). This theory posits that when adolescents do not cultivate positive peer relationships in reality and their needs for connection and belonging remain unfulfilled, they often retreat into the virtual realm of the Internet to find a sense of belonging and security, thereby substantially heightening their risk of IA. A cross-sectional study of 318 high school students revealed that negative emotions increase following peer rejection, prompting adolescents to engage more actively or passively with social media as a means of emotional compensation (Zhao et al., 2024). Consequently, we hypothesized that adolescents encountering PV may rely on the Internet as an escape from reality, thereby increasing their susceptibility to IA.

### 1.3 The mediating role of SE

SE pertains to an individual’s appraisal of their performance in social roles, including positive assessments and feelings of self-worth derived from social comparison (Orth and Robins, 2022). High SE diminishes the risk of adverse emotions, improves self-regulation capabilities, and facilitates the systematic development of psychological wellbeing, establishing it as a crucial psychological protective factor (Henriksen et al., 2017). Conversely, individuals with low SE demonstrate considerable psychological susceptibility and are major risk factors for the emergence of multifaceted psychological disorders. The Interaction of Person-Affect-Cognition-Execution (I-PACE) model asserts that IA arises from the interplay of personal traits, internal and external stimuli, emotional regulation, and cognitive processes, with these factors potentially acting as mediators (Brand et al., 2019). Poor peer relationships serve as external catalysts for IA, but SE constitutes a fundamental human characteristic that embodies adolescents’ self-identity. Current research has validated its strong association with both peer relationships and IA. Consequently, SE may act as a bridge between poor peer relationships and IA.

Peers are among the social agents most intimately linked to teenagers, and positive peer relationships work as a protective factor for the development of adolescent SE (Khan et al., 2023). Research reveals that when individuals perceive acceptance and affection in significant relationships, such as among peers, their SE increases; conversely, rejection and exclusion lead to a drop in SE (Zeng et al., 2025). Moreover, the cognitive-behavioral model of pathological Internet usage identifies SE as a crucial determinant in IA (Zeng et al., 2023). Zhang et al. conducted a study involving 1,068 Chinese undergraduates, revealing that SE affects individuals’ ability to self-regulate: those with lower SE are more prone to external influences that compel them to act contrary to their wishes, while individuals with higher SE are less likely to forfeit behavioral control (Zhang et al., 2015). In reality, individuals with low SE frequently experience dissatisfaction and a deficiency of confidence; their basic psychological needs for belonging and self-worth are unfulfilled (Zhang et al., 2024), while the Internet provides a relatively anonymous setting that circumvents immediate exposure to negative feedback, facilitating their immersion. A study involving 257 Spanish adolescents indicated that improved peer relationships can indirectly and significantly diminish the prevalence of IA by positively influencing adolescents’ SE (Wang et al., 2017). Furthermore, Xu et al. (2023) demonstrated in a sample of 830 adolescents that peer pressure may indirectly elevate the risk of social media addiction by lowering SE. Taken together, we propose hypothesis that SE may partially mediate the association between PV and IA.

### 1.4 The moderating role of TSR

While PV is a significant risk factor connecting low SE and IA in adolescents, not all youths are uniformly impacted by PV. Consequently, it is essential to investigate the moderators of the relationships among PV, SE, and IA. Bronfenbrenner’s ecological systems theory asserts that school serves as a fundamental microsystem for adolescents, encompassing linked subsystems such as peer and teacher-student relationships (Bronfenbrenner and Ceci, 1994). When one subsystem is compromised, another may compensate by enhancing its performance (Ahrendt et al., 2018). TSR is a fundamental protective factor for adolescent mental health: a positive relationship offers emotional support, fulfills adolescents’ affiliation needs, strengthens self-identity, and moderates negative emotions, thereby significantly impacting adolescent mental health (Guo et al., 2019).

Positive TSR can markedly bolster adolescents’ resilience to negative influences. A study including 69 teachers and 2,147 students indicated (Hu et al., 2024) that the professional identity of novice teachers enhances TSR, thus bolstering adolescents’ psychological resilience. Psychological resilience denotes the ability and dynamic process through which individuals maintain normal psychological functioning, uphold favorable developmental paths, or efficiently recover and adapt when faced with substantial adversity, trauma, stress, or risk conditions (e.g., PV). A cross-sectional study including 352 teenagers revealed that the positive TSR strongly predicted elevated emotional intelligence, defined as the capacity to perceive, understand, express, and regulate one’s own and others’ emotions successfully (Wan et al., 2023). Adolescents possessing elevated emotional intelligence exhibit more awareness of their own and others’ emotions, enabling them to express and regulate their emotions more proficiently, thereby alleviating the effects of adverse occurrences. Thus, in confronting bad experiences like PV, a better TSR may assist adolescents in resisting negative influences by bolstering psychological resilience and emotional intelligence, thereby mitigating the harmful consequences of PV on SE and its facilitative role in IA (Cornelius-White, 2007). A meta-analysis has revealed that current research on adolescent IA frequently examines the effects of parent–child, peer, or teacher–student relationships in isolation, with limited investigation into their combined or interacting effects. This study posits that TSR moderates the association between PV and SE, as well as the direct influence of PV on IA. A positive TSR may mitigate the predicted effects of PV on low SE and IA.

**Figure 1:**
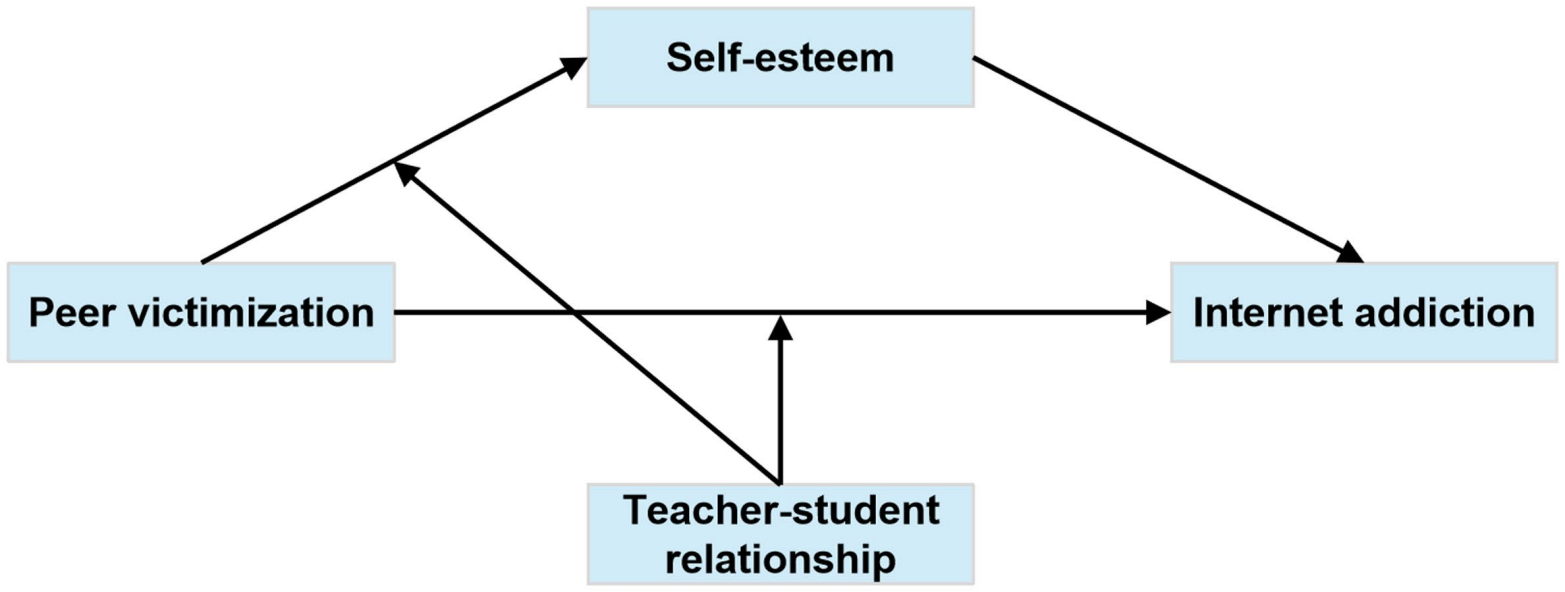
Hypothesized conceptual model of the moderated mediation.

In summary, this study seeks to investigate the mediating role of SE in the relationship between PV and adolescent IA, alongside the moderating influence of TSR within this mediation framework. A moderated mediation model is developed to investigate the mediating and moderating mechanisms by which PV affects adolescent IA, based on the research hypotheses.(See the conceptual model in Figure 1).

## 2 Method

### 2.1 Participants and procedure

Using convenience sampling, 1,162 students from six high schools in Jiangsu Province were recruited as participants. The demographic characteristics were as follows: gender (42.51% male, 57.49% female), grade level (35.63% Grade 10, 46.56% Grade 11, 17.81% Grade 12), family structure (53.79% only child, 46.21% non-only child), residential area (95.27% urban, 4.73% rural), with a mean age of 15.8 ± 0.74 years.

The study protocol received approval from the appropriate institutional authority. With the cooperation of classroom teachers, trained research assistants entered the classrooms to administer the survey. Participants were informed that their involvement was entirely voluntary and that their responses would be treated anonymously and confidentially. Next, the research assistants handed out paper-based questionnaires, which included measures of PV, TSR, SE, and IA. On average, participants completed the questionnaire in approximately 10 min. Once finished, the questionnaires were collected in a standardized manner by the research assistants.

### 2.2 Measures

#### 2.2.1 PV

This study employed the Multidimensional Peer Victimization Scale (MPVS) developed by Mynard and Joseph (Betts et al., 2015) to assess the extent of PV among participants. The questionnaire consists of 11 items, requiring adolescents to report the frequency of experiencing physical and verbal victimization by peers over the past 6 months. Responses are rated on a 4-point Likert scale, ranging from 1 (“never”) to 4 (“very often”). A total score is computed by summing all items, with higher scores indicating greater levels of PV. In the present study, Cronbach’s *α* for the physical and relational victimization subscales were 0.770 and 0.879, respectively. The Cronbach’s *α* is 0.879 for the overall scale.

#### 2.2.2 TSR

This study employed the TSR Scale (STRS), developed by Zou et al. (2007) based on the research of Wang Yun et al., to assess TSR. The questionnaire consists of 23 items, measuring four dimensions: positive intimacy (the degree of closeness perceived by students in their relationship with teachers), supportiveness (the extent of support perceived by students from teachers), satisfaction (students’ satisfaction with their relationship with teachers), and negative conflict (the degree of conflict perceived by students in their relationship with teachers). Items are rated on a 5-point Likert scale, ranging from 1 (“strongly disagree”) to 5 (“strongly agree”). Negative conflict was reverse-scored, and a total score was calculated by summing all items, with higher scores indicating better TSR. In the present study, Cronbach’s *α* for intimacy, supportiveness, satisfaction, and conflict were 0.891, 0.851, 0.799, and 0.874, respectively. The Cronbach’s *α* is 0.966 for the overall scale.

#### 2.2.3 SE

This study employed the Rosenberg Self-Esteem Scale (RSES) to measure adolescent SE levels. The scale consists of 10 items, such as “I feel that I am a person of worth, at least on an equal plane with others” and “I have a positive attitude toward myself.” Responses are rated on a 4-point Likert scale, ranging from 1 (“strongly disagree”) to 4 (“strongly agree”). A total score is calculated by summing all items, with reverse scoring applied to items 3, 5, 8, 9, and 10. Higher total scores indicate higher SE. In the present study, the Cronbach’s *α*, was 0.902 for the scale.

#### 2.2.4 IA

This study employed the Chinese Internet Addiction Scale–Revised (CIAS-R), developed by Chen Shuhui et al. based on the original Chinese Internet Addiction Scale (CIAS), to assess adolescents’ levels of IA. The scale comprises 26 items divided into five dimensions: IA tolerance, compulsive Internet use, Internet withdrawal reaction, interpersonal and health problems, and time management problems over the past 6 months. Items are rated on a 4-point Likert scale, ranging from 1 (“strongly disagree”) to 4 (“strongly agree”). A total score is computed by summing all items, with higher scores indicating greater severity of IA. In the present study, Cronbach’s *α* for addiction tolerance, compulsive internet use, internet withdrawal reaction, interpersonal and health problems, and time management problems are 0.786, 0.889, 0.863, 0.923, and 0.754, and for the overall scale was 0.966.

### 2.3 Data analyses

Data entry, cleaning, and preliminary statistical analyses were performed using Microsoft Excel. To examine the direct and indirect effects of PV on IA and the moderating role of TSR in this association, both a simple mediation model and a moderated mediation model were tested using the PROCESS macro for SPSS version 29.0. The bias-corrected percentile bootstrap method with 5,000 resamples and 95% confidence intervals was employed to estimate indirect effects and test their statistical significance. Control variables were included in all models, and each path coefficient was examined step by step following the hierarchical regression procedure to evaluate the incremental explanatory power of the mediating and moderating effects.

## 3 Results

### 3.1 Common method bias test

Harman’s single-factor test was conducted to examine the potential common method bias among the four variables (PV, TSR, SE, and IA). The results revealed that nine factors had eigenvalues greater than one, and the first factor accounted for 21.704% of the total variance, which is below the critical threshold of 40%. This indicates that common method bias was not a significant concern in the present study. The cumulative variance explained by all factors was 63.122%, suggesting that the overall model fit was acceptable.

Previous studies have identified gender, age, boarding status, frequency of conflict with parents, and parental educational level as significant factors influencing adolescents’ SE and IA (Yen et al., 2009; Li et al., 2014). Therefore, these demographic variables were included as control variables in all subsequent analyses.

### 3.2 Correlation analysis among variables

Table 1 presents the means, standard deviations, and correlation coefficients for PV, TSR, SE, and IA. The results indicated that PV was significantly and positively correlated with IA, suggesting that PV is an important risk factor for IA. SE was significantly negatively correlated with both PV and IA, supporting the mediating role of SE in the association between PV and IA. In addition, the teacher–student relationship was positively associated with SE and negatively associated with IA, providing preliminary evidence for its moderating role in the indirect pathway of “PV → SE → IA.”

**Table 1:** Correlations between PV, TSR, SE, and IA.

Variables	M	SD	PV	TSR	SE	IA
PV	13.028	3.61	1			
TSR	38.874	14.079	−0.330***	1		
SE	19.197	5.537	−0.331***	0.580***	1	
IA	48.621	13.868	0.288***	−0.491***	−0.501***	1

M, mean; SD, standard deviation. *p < 0.05, **p < 0.01, ***p < 0.001.

Linear regression analyses of IA, PV, teacher–student relationship, and SE yielded Variance Inflation Factor (VIF) values of 1.124, 1.482, and 1.423, respectively, all below the conventional threshold of 5, indicating no multicollinearity issues.

### 3.3 Mediation effect of SE

As shown in Table 2, the 95% bootstrap confidence intervals for all path coefficients did not include zero, indicating a significant mediating effect of SE. Specifically, PV had both a direct effect on IA and an indirect effect through SE. The direct effect (0.667) and indirect effect (0.336) accounted for 66.5 and 33.5% of the total effect (1.003), respectively, suggesting that SE partially mediates the relationship between PV and IA.

**Table 2:** Path analysis of chain mediation model.

Model pathways	Effect value	SE	95%CI		Effect ratio
			LLCL	ULCL	
Total effect	1.003	0.108	0.792	1.215	
Direct effect	0.667	0.101	0.47	0.864	66.50%
Indirect effect	0.336	0.044	0.251	0.424	33.50%

SE, standard error (elsewhere in the manuscript, SE refers to self-esteem).

### 3.4 Moderation effect of TSR

In addition to examining the mediating role of SE in the relationship between PV and IA, this study also investigated the moderating effect of TSR on the mediating pathway. As shown in Table 3, both PV (*b* = −0.332, SE = 0.059, *β* = −0.162, *p* < 0.001) and TSR (*b* = 0.236, SE = 0.013, *β* = 0.487, *p* < 0.001) significantly predicted SE. Moreover, the interaction term between PV and TSR also significantly predicted SE (*b* = −0.018, SE = 0.003, *β* = −0.010, *p* < 0.001). Furthermore, SE (*b* = −0.548, SE = 0.060, *β* = −0.274, *p* < 0.001) and TSR (*b* = −0.236, SE = 0.028, *β* = −0.266, *p* < 0.001) were both significant predictors of IA; however, the interaction between SE and TSR (*b* = −0.004, SE = 0.004, *β* = −0.010, *p* > 0.05) did not significantly predict IA. Finally, PV (*b* = 0.633, SE = 0.116, *β* = 0.162, *p* < 0.001) significantly predicted IA, and the interaction term between PV and TSR (*b* = 0.014, SE = 0.006, *β* = 0.054, *p* < 0.05) also reached statistical significance. Taken together, these results suggest that TSR moderates both the first stage of the mediating pathway (“PV → SE → IA”) and the direct effect of PV on IA.

**Table 3:** Regression analysis results for the moderated mediation model.

Regression equation		Model fit statistics			Regression coefficients (*b*) and significance levels			
Outcome variable	Predictive variable	VIF	*R* ^2^	*F*	*b*	SE	*β*	*t*
		0.587	0.345	67.314***				
SE	PV				−0.332	0.059	−0.162	−5.596***
	TSR				0.236	0.013	0.487	18.315***
	PV*TSR				−0.018	0.003	−0.010	−6.138***
		0.585	0.342	54.273***				
IA	PV				0.633	0.116	0.162	5.482***
	SE				−0.548	0.060	−0.274	−9.212***
	TSR				−0.236	0.028	−0.266	−8.402***
	PV*TSR				0.014	0.006	0.054	2.330*
	TSR*SE				−0.004	0.004	−0.010	−0.122

PV, peer victimization; TSR, teacher–student relationship; SE, self-esteem; IA, internet addiction;*p < 0.05, **p < 0.01, ***p < 0.001.

### 3.5 Simple slope analysis

**Figure 2:**
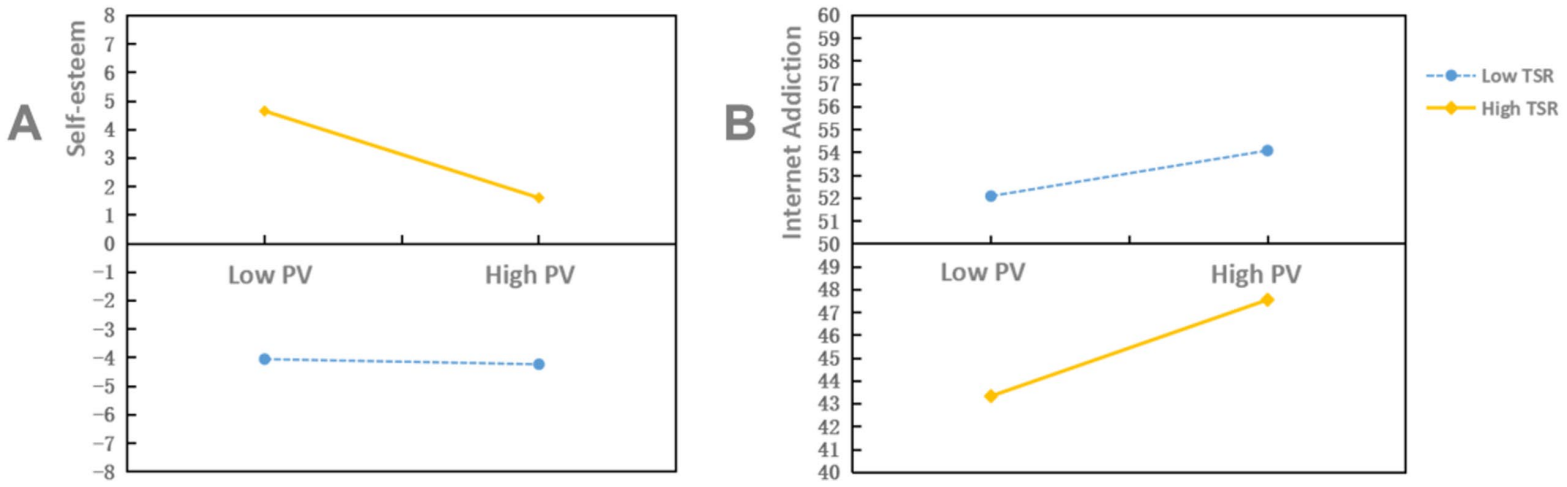
The moderating role of TSR between PV and both SE and IA. **(A)** The moderating role of TSR between PV and SE; **(B)** The moderating role of TSR between PV and IA; high PV one standard deviation above the mean (M + SD); low PV one standard deviation below the mean (M − SD).

To further explore the moderating effect of TSR, a simple slope analysis was conducted, as depicted in Figure 2. for participants with a high level of TSR (M + SD), PV significantly predicted SE (simple slope = −0.593, *t* = −6.627, *p* < 0.001); whereas for those with a low level of TSR (M − SD), PV did not significantly predict SE (simple slope = −0.056, *t* = −1.052, *p* = 0.293). In addition, for participants with a high level of TSR (M + SD), PV significantly predicted IA (simple slope = 1.158, *t* = 6.494, *p* < 0.001). For participants with a low level of TSR (M − SD), PV also significantly predicted IA (simple slope = 0.438, *t* = 4.124, *p* < 0.001); however, the predictive association was substantially weaker.

## 4 Discussion

### 4.1 The relationship between PV and IA

The current study found a significant positive relationship between PV and IA, implying that PV is an important risk factor for teenagers’ problematic online behaviors. Adolescents who experience PV frequently report elevated levels of anxiety and depression. Stress-coping theory suggests that such negative emotions may predispose them to use maladaptive coping strategies—such as excessive Internet use—to alleviate distress and escape from real-world stressors (Sullivan et al., 2006; Kliewer et al., 2012) Furthermore, adolescents exposed to PV have lower levels of peer connectivity and less possibilities for positive interpersonal engagement. On the one hand, limiting interaction with peers limits the development of self-efficacy, which may increase the chance of maladaptive behaviors like IA (Saw et al., 2002). On the other side, the lack of satisfying real-life interactions may leave adolescents’ psychological needs unsatisfied, leading them to seek emotional attachment and social support in virtual environments, increasing the risk of IA.

Within the adolescent socioecological framework and in accordance with ecological systems theory, the family—an integral component of the microsystem—is critical and irreplaceable in defining behavioral trajectories and psychological adjustment during adolescence. Its influence spreads through dynamic interactions with mesosystem elements as peer contexts. Post-hoc analyses revealed that the control variable “frequency of conflicts with parents” substantially predicted both SE (*b* = −1.425, *p* < 0.001) and IA (*b* = 1.307, *p* < 0.05). This corresponds with previous studies (Wang et al., 2014; Zhou et al., 2022; Liu and Kuo, 2007). Parent–child and peer relationships are two interrelated parts of adolescents’ social networks, therefore their mutual influences are critical for understanding youth psychological and behavioral outcomes (Qin et al., 2025). Future research should investigate the combined influence of various relational systems on adolescents IA and construct an integrated social network model to better understand the interpersonal dynamics that underpin its development.

### 4.2 The mediating role of SE

The data suggested that SE acted as a mediator in the relationship between PV and IA. Exposure to PV was related with lower SE, and latter was associated with a greater proclivity for maladaptive online interaction like IA. Adolescents with more favorable peer interactions, on the other hand, reported higher SE, which was associated with a lower risk of IA. These findings are consistent with previous studies. Peers have an important role in adolescents’ interpersonal interactions because school is their primary developmental environment. When adolescents have PV, their sense of self-worth tends to deteriorate, and basic psychological needs might remain unmet. Such experiences can increase the tendency to detach from painful real-life circumstances and seek emotional compensation in online platforms, raising the risk of IA. Adolescents who retain supportive peer relationships and receive positive social feedback, on the other hand, tend to report higher SE, which may improve self-regulation and minimize vulnerability to IA.

Beyond identifying the mediating role of SE, the present study also highlighted a robust association between SE and IA. SE is a key psychological resource supporting adolescents’ mental health and resilience, whereas IA represents a salient behavioral risk factor during this developmental period. Evidence suggests that the two may influence each other over time. A two-year longitudinal study involving 1,736 adolescents (Lai et al., 2023) demonstrated that baseline low SE significantly predicted Pathological Internet Use (PIU, a wider category of problematic use, of which IA reflects the more severe and addiction-like end). One year later, while baseline PIU also significantly predicted lower SE at the subsequent follow-up. This reciprocal association suggests a potential maladaptive cycle in which “low SE → PIU → further reductions in SE” may be reinforced over time. Such bidirectional findings deepen the understanding of the broader “PV–SE–IA” pathway and indicate that future work may benefit from applying longitudinal network approaches to elucidate how intervention-related variables, SE, and IA interact dynamically over time.

From a practical perspective, these findings highlight the value of developing dual-target intervention frameworks. Specifically, psychological counseling may help adolescents with low SE strengthen self-concept and emotion management, while digital literacy programs may support healthier online behavior patterns. Interventions addressing both psychological vulnerabilities and behavioral regulation may be especially effective in disrupting the maladaptive cycle between SE and IA, providing a more targeted strategy for preventing IA in adolescence.

### 4.3 The moderating role of TSR

The study demonstrated that TSR had both direct and indirect moderating effects on IA in adolescents. First, the predictive association between PV and IA was stronger among adolescents with higher levels of TSR, indicating that supportive TSR may attenuate the risk of IA for those exposed to PV. Adolescents who experience positive TSR are more likely to receive emotional support, affirmation, and a sense of belonging from their interactions with teachers, which may partially mitigate the psychological distress linked to PV and diminish their inclination to seek escape or emotional relief through excessive Internet use. Second, TSR also moderated the negative correlation between PV and SE. Adolescents with higher levels of TSR may receive more positive feedback, and develop better psychological resilience and emotional competence. These resources may subsequently diminish the correlation between PV and IA by promoting the maintenance of SE.

*Post hoc* analyses revealed a differentiated pattern across TSR dimensions: the “support” dimension moderated both the first half of the mediation pathway (*b* = −0.080, *p* < 0.001) and the direct association between PV and IA (*b* = 0.079, *p* = 0.009), whereas the “intimacy” dimension moderated only the first half of the mediation pathway (*b* = −0.042, *p* < 0.001) and did not significantly moderate the direct pathway (*b* = 0.024, *p* = 0.140). These findings imply that intervention strategies should be tailored to students’ specific profiles. For adolescents exposed to PV who exhibit lowered SE but do not yet show IA tendencies, teachers may emphasize both intimacy and support by engaging in proactive communication and providing affirming feedback to foster SE. For adolescents whose SE remains relatively intact but who display elevated IA tendencies, teachers may prioritize the “support” dimension by guiding healthy online behavior and offering alternative real-world social engagement. For adolescents experiencing both diminished SE and IA tendencies, an integrated approach involving both intimacy and support may be most beneficial, simultaneously strengthening self-worth and guiding adaptive Internet use.

The preceding section has emphasized that SE mediates the relationship between PV and IA, and that SE and IA may establish a maladaptive cycle over time. TSR may diminish the predicted associations between PV and both SE and IA, therefore offering a means to disrupt this cycle. For adolescents consistently exposed to PV, early identification and targeted intervention by teachers are crucial. One-on-one counseling may help rebuild SE by offering individualized emotional support, whereas implementing digital literacy programs in the class may guide students toward healthier online practices. These measures may interrupt the mutual reinforcing of low SE and IA, hence improving intervention efficacy. Collectively, the data indicate that TSR serves as a protective factor for adolescent IA by modulating the relationship between PV and IA, either directly or by affecting the mediator SE.

## 5 Implication

This study provides several theoretical and practical insights for understanding and tackling IA among adolescents in relation to PV and TSR. The study theoretically integrates multiple frameworks to develop a moderated mediation model, utilizing the Compensatory Internet Use Theory to elucidate the relationship between PV and increased IA tendencies, employing the I-PACE framework to delineate the mediating role of SE, and incorporating ecological systems theory to emphasize the moderating effect of TSR. Collectively, these viewpoints delineate a moderated mediation model that elucidates the framework of the “PV → SE → IA” pathway. Furthermore, by clarifying reciprocal relationships between SE and IA, the study transcends static mediation models and offers a dynamic viewpoint that highlights the cyclical interaction between lower SE and IA.

Practically, the findings offer practical guidance for IA intervention in adolescents. First, adolescents exposed to PV constitute a high-risk demographic, and early detection can provide targeted psychological assistance to reduce IA susceptibility. Second, the exhibited buffering capacity of TSR indicates that school-based interventions could reinforce this protective factor via teacher training focused on improving relational closeness, supportive communication, and conflict-resolution abilities. Third, in view of the reciprocal pattern between low SE and IA, a dual-target strategy—combining psychological counseling to increase SE with digital-literacy programs to prevent IA—may offer a practical approach for breaking maladaptive cycles and enhancing intervention effectiveness.

## 6 Limitation

Several constraints must be recognized. First, although the cross-sectional design reduces concerns regarding common method bias, it does not permit clear temporal ordering among variables and therefore limits causal inference (for instance, it remains uncertain whether PV precedes reductions in SE or whether adolescents with lower SE are more susceptible to PV), which prevents the examination of dynamic developmental trajectories. Second, the sample’s representativeness is limited, as participants were sourced from six high schools within a single province in Jiangsu, focusing solely on high school students and excluding junior high or university populations. This limited sampling frame constrains the generalizability of the findings. Third, the study did not examine potential grade-level or gender disparities in the moderating role of TSR, and future research may further explore how TSR functions across various subgroups. Finally, measurement limitations should be considered, as core constructs (PV, SE, IA, TSR) were assessed solely through self-report instruments. While these scales shown acceptable reliability, responses may be affected by social desirability or recall biases.

## 7 Conclusion

This study integrated the Compensatory Internet Use Theory, the I-PACE framework, and ecological systems theory to examine the associations between PV and IA, as well as the mediating role of SE and the moderating role of TSR. First, PV, as a prominent factor associated with negative psychological consequences in adolescence, demonstrated a substantial positive correlation with IA. Second, SE, as an essential resource supporting self-coherence and psychological adjustment, served as a mediator between PV and IA. Finally, TSR, as a key protective factor in adolescent mental health, moderated both the first stage of the “PV → SE → IA” pathway and the direct association between PV and IA. Higher levels of TSR exacerbated the negative correlation between PV and SE while reinforcing the predictive relationship between PV and IA.

## Data Availability

The raw data supporting the conclusions of this article will be made available by the authors, without undue reservation.
